# In this issue of *Current Oncology*

**Published:** 2007-12

**Authors:** M. McLean

Although the last issue of *Current Oncology* contained a consensus guideline on the management of anemia in cancer, pressure on our peer review process led to an inability to include a timely related manuscript from Dr. Amanda Goldrick and colleagues. I am pleased to rectify that situation in the present issue. In a retrospective review covering 2002–2003, Dr. Goldrick’s group highlights the relatively low rate of physician intervention in more than 700 cases in which breast cancer patients being treated in the adjuvant setting became significantly anemic as a result of their therapy. The rate of intervention involving a discussion (for example) with patients whose hemoglobin levels were below 90 g/L was less than half. Nowadays, we realize that a number of studies describe quality-of-life benefits for patients who receive support.

Our practice guidelines series continues with an update on oxaliplatin funding in the management of gastrointestinal malignancy from Dr. Derek Jonker, of the Gastrointestinal Cancer Disease Site Group of Cancer Care Ontario’s Program in Evidence-Based Care.

A short communication from Dr. Max Dahele and his colleagues outlines suggested newer management options for lung cancer, featuring integration in management strategies by community hospitals and cancer referral centres in Ontario. The authors promise an update once these policies have been fully implemented and tested.

Our guest editorials include a review of the antitumour activities of statins from Drs. Takahashi and Nishibori, and a detailed discussion of the role of vaccine therapy in cancer from Dr. Jeffrey Schlom and colleagues.

A report—a first for *Current Oncology*—from the recent Gairdner Award lectures is likewise included in this issue. The year 2007 marks the 48th presentation of the Gairdner Awards, one quarter of whose past 288 recipients have gone on to win a Nobel Prize (including two Nobel Laureates presenting lectures at this year’s symposium).

As 2007 draws to a close, I would like again, as Editor, to express my gratitude on behalf of the editorial board for your submissions, the efforts of our publisher, and the support of industry, without whom we would be unable to continue our work.

